# Development and Validation of EST-SSR Markers from the Transcriptome of Adzuki Bean (*Vigna angularis*)

**DOI:** 10.1371/journal.pone.0131939

**Published:** 2015-07-06

**Authors:** Honglin Chen, Liping Liu, Lixia Wang, Suhua Wang, Prakit Somta, Xuzhen Cheng

**Affiliations:** 1 The National Key Facility for Crop Gene Resources and Genetic Improvement, Institute of Crop Science, Chinese Academy of Agricultural Sciences, Beijing, 100081, China; 2 State Key Laboratory of Agrobiotechnology, College of Biological Sciences, China Agricultural University, Beijing, 100193, China; 3 Department of Agronomy, Faculty of Agriculture at Kamphaeng Saen, Kasetsart University, Nakhon Pathom, 73140, Thailand; National Institute of Plant Genome Research, INDIA

## Abstract

The adzuki bean (*Vigna angularis* (Ohwi) Ohwi and Ohashi) is an important grain legume of Asia. It is cultivated mainly in China, Japan and Korea. Despite its importance, few genomic resources are available for molecular genetic research of adzuki bean. In this study, we developed EST-SSR markers for the adzuki bean through next-generation sequencing. More than 112 million high-quality cDNA sequence reads were obtained from adzuki bean using Illumina paired-end sequencing technology, and the sequences were *de novo* assembled into 65,950 unigenes. The average length of the unigenes was 1,213 bp. Among the unigenes, 14,547 sequences contained a unique simple sequence repeat (SSR) and 3,350 sequences contained more than one SSR. A total of 7,947 EST-SSRs were identified as potential molecular markers, with mono-nucleotide A/T repeats (99.0%) as the most abundant motif class, followed by AG/CT (68.4%), AAG/CTT (30.0%), AAAG/CTTT (26.2%), AAAAG/CTTTT (16.1%), and AACGGG/CCCGTT (6.0%). A total of 500 SSR markers were randomly selected for validation, of which 296 markers produced reproducible amplicons with 38 polymorphic markers among the 32 adzuki bean genotypes selected from diverse geographical locations across China. The large number of SSR-containing sequences and EST-SSR markers will be valuable for genetic analysis of the adzuki bean and related *Vigna* species.

## Introduction

The adzuki bean (*Vigna angularis* (Ohwi) Ohwi and Ohashi) is a diploid crop (2n = 2x = 22) with a genome size of approximately 500 Mb. It is an important temperate legume, with high nutritional value, grown for human consumption. Adzuki bean has been widely grown in East Asian countries, especially in China, Japan and Korea, for thousands of years [1]. In these countries, the adzuki bean is the second-most important legume crop after soybean. The crop is also grown to some extent in Bhutan, Nepal, and India [2]. Adzuki bean seeds are generally used to make desserts or pastry filling because of their natural sweetness and great taste. China has a long history of growing adzuki bean and is considered a center of origin of this crop. Abundant germplasm resources of adzuki bean are present in China, which is the largest producer of adzuki bean, with an annual production of approximately 300,000 metric tons.

However, molecular breeding of adzuki bean has lagged behind that of other legume crops such as soybean and common bean because of the lack of genomic resources. Molecular markers are an important genetic tool for gene mapping and marker-assisted selection (MAS) for crop improvement. At present, the simple sequence repeat (SSR; also known as microsatellite) and the single nucleotide polymorphism (SNP) are the standard DNA markers of choice for gene mapping and MAS in many crops. SNP and SSR markers share similar advantages including being co-dominant, abundant throughout genome and highly polymorphic. However, SSRs are multi-allelic, while most cases of SNPs are bi-allelic. In addition, SNP detection is difficult and needs expensive machinery, while detection of SSRs can be carried out using standard PCR and gel electrophoresis. In contrast to the genomic SSRs, EST-SSRs are located in the coding region of the genome and have some intrinsic advantages. For example, they can be quickly obtained by electronic sorting and are highly transferable to related taxa. Because of these advantages, EST-SSRs have been developed and used in many plant species. Although a major disadvantage of the EST-SSR is the sequence redundancy that yields multiple sets of markers at the same locus, this problem can be circumvented by assembling the ESTs into a unigenes. There are few reports on SSR markers for adzuki bean to date. A total of 50 genomic SSR markers and 1,429 EST-SSR markers were developed in adzuki bean [3, 4]. However, the number of SSR markers reported for adzuki bean is still far fewer than those reported for other legumes such as common bean [5, 6], chickpea [7], pigeon pea [8] and soybean [9, 10].

Recent advances in next-generation sequencing (NGS) technologies enable the generation of massive amounts of nucleotide sequences efficiently and cost-effectively. NGS makes whole-transcriptome sequencing (RNA sequencing; RNA-seq) and analysis in crops feasible [11]. Whole-transcriptome sequencing is an effective approach for functional gene discovery and for insights into the expression and regulation networks of genes [12–14]. It is also useful for identifying and developing large numbers of EST-SSR markers for crops, especially minor crops that lack genomic resources.

In this study, a large number of high-quality transcriptome sequences were generated and a large number of EST-based SSRs was developed for adzuki bean using the Illumina HiSeq 2000 platform. We characterized the distribution of SSR motifs in the sequences generated and validated a set of EST-SSR for further use in diversity analysis. We also discuss the utility of these microsatellite markers for comparative mapping.

## Materials and Methods

### Plant materials

In total, 34 varieties or accessions of adzuki bean were used. These adzuki bean accessions were obtained from the National Center for Crop Germplasm Resources Preservation, Institute of Crop Science, Chinese Academy of Agricultural Sciences, Beijing, China. Two varieties, ‘Zhonghong5’ and ‘Jingnong2’, were used for RNA-seq. Thirty-two accessions, collected from various parts of China, were used for genetic diversity analysis (S1 Table).

### RNA isolation and cDNA library construction

Total RNA was isolated from the roots, stems, leaves and flowers of five plants of each adzuki bean variety using Trizol Reagent, according to the manufacturer’s instructions (Invitrogen, Carlsbad, CA, USA). The RNA was then treated with RNase-free DNase I (Takara, Otsu, Shiga, Japan) at 37°C for 30 min to remove residual DNA. RNA quality was verified using a 2100 Bioanalyzer (Agilent Technologies, Santa Clara, CA) and checked by RNase-free agarose gel electrophoresis. The concentration of the total RNA was further quantified using NanoDrop 2000 (Thermo Fisher Scientific, Wilmington, Delaware USA). Equal amounts of total RNA from each adzuki bean varieties were quickly frozen in liquid nitrogen for storage at -80°C until further use.

A cDNA library of each pooled RNA was obtained using a TruSeq RNA Sample preparation kit (Illumina, USA), according to the manufacturer's instructions. Subsequently, equivalent amounts of total cDNA from the two different adzuki bean varieties were mixed together. Poly-T oligo-attached magnetic beads (Illumina Inc., San Diego USA) were used to isolate poly-A mRNA from total RNA. First-strand cDNA was synthesized from the fragmented mRNA using random hexamer primers and reverse transcriptase (Invitrogen, USA). The single-end cDNA library was prepared in accordance with Illumina’s protocol.

### Illumina Sequencing, data filtering and *de novo* assembly

The cDNA library was sequenced using PE90 strategy with Illumina paired-end sequencing technology according to the standard Illumina protocol in the Beijing Genome Institute (Shenzhen, China) [15]. The libraries were sequenced in one lane then raw-reads were sorted out by barcodes, and the data were automatically collected and generated into FASTQ files (.fq) containing raw data for all the reads. The data have been submitted to the Sequence Read Archive (SRA) of the NCBI database under accession SRP049807. The raw sequence data were first cleaned by trimming adapter sequences. Reads containing more than 10% of bases with a poor quality score (Q<20), non-coding RNA, ambiguous sequences containing an excess of “N” nucleotide calls or adaptor contamination were removed. The reads that did not pass the Illumina failed-chastity filter were discarded, according to “failed-chastity ≤ 1” with a threshold of 0.6 on the first 25 cycles. *De novo* transcriptome assembly was then performed with Trinity [16].

### Unigene annotation

The unigenes were aligned with BLASTX against the NCBI non-redundant (NR) proteins [17, 18]. The proteins with highest sequence similarity were retrieved and annotated to each unigene. With nucleotide based annotation, Blast2GO [19] software was used to obtain GO annotation categories defined by molecular function, cellular component and biological process ontologies.

### SSR search and primer design

MISA (MIcroSAtellite; http://pgrc.ipk-gatersleben.de/misa) and SAMtools [20] were employed for SSR mining and identification. The minimum number of repeats used for selecting the SSRs was ten for mono-nucleotide repeats, six for di-nucleotide repeats, five for tri-nucleotide repeats, and three for tetra-, penta-, and hexa-nucleotide repeats. Primers for SSRs were designed using Premier 5.0 (PREMIER Biosoft International, Palo Alto, California, USA) with the following criteria: primer lengths of 16–22 bases, GC content of 40–60%, annealing temperature of 40°C-60°C, and PCR product size of 100 to 300 bp.

### Marker validation

A total of 500 EST-SSR markers were validated using 32 adzuki bean accessions (S1 Table). Genomic DNA of each adzuki bean accession was extracted from young leaves using the Hexadecyl trimethyl ammonium Bromide (CTAB) method [21]. The quality and quantity of DNA was evaluated on a 1% agarose gel. The DNA concentration was adjusted to 50 ng/μl. PCR was performed in a total volume of 20 μl containing 50 ng of genomic DNA, 0.5 U of Taq DNA polymerase (Dingguo Biological Technology Development Co., Ltd, Beijing, China), 1× of Taq Buffer II, 1.5 mM MgCl_2_, 25 μM of dNTPs, and 0.4 μM each forward and reverse primer. PCR amplification was carried out using a Heijingang Thermal Cycler (Eastwin, Beijing, China) with the following cycling conditions: pre-denaturation at 94°C for 4 min followed by 30–35 cycles of 94°C for 30 sec, 55–60°C (depending on primers) for 30 sec and 72°C for 30 sec, and finally, 5 min at 72°C. The PCR products were separated on an 8.0% non-denaturing polyacrylamide gel electrophoresis (PAGE) gel and then visualized by silver staining. The pBR322 Marker I DNA ladder (Zheping, Biological Technology Development Co., Ltd, Beijing, China) was used as the standard size marker.

### Genetic diversity analysis

The number of alleles (Na), observed heterozygosity (H_o_), gene diversity (expected heterozygosity; He), and polymorphism information content (PIC) for each of the EST-SSR markers were calculated using PowerMarker V3.25 [22]. A genetic similarity matrix based on the “proportion of shared alleles” among the 32 adzuki bean accessions was generated using PowerMarker. An unrooted neighbor-joining tree based on the shared allele distances was constructed using MEGA 4 software [23] to reveal genetic relationships among the 32 adzuki bean accessions.

## Results

### Sequencing and *de novo* assembly of Illumina paired-end reads from adzuki bean transcriptomes

**Fig 1:**
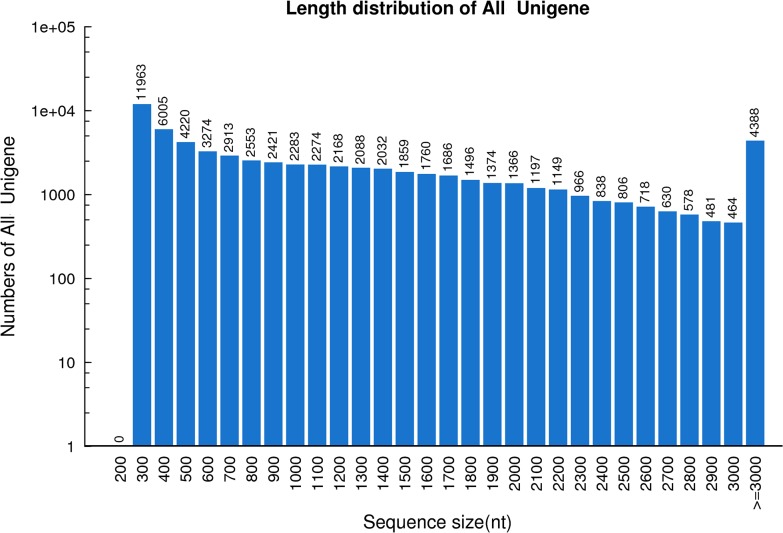
Frequencies length distribution of Illumina read sequences.

A total of 54.79 and 57.24 million paired-end raw reads were obtained for the ‘Jingnong2’ and ‘Zhonghong5’ varieties, respectively. After removal of the low-quality reads, 52.11 and 54.30 million clean reads with GC content of 45.9% and 46.3% were obtained for the ‘Jingnong2’ and ‘Jinghong5’ varieties, respectively. The sequence quality, based upon the clean reads of both adzuki bean varieties, was 98.4% of Q20. The combined sequences of these reads were assembled into 65,950 unigenes by Trinity. The average length of the unigene is 1,213 bp (N50 = 1,889 bp). The lengths of the unigenes ranged from 200 to 19,090 bp. Of these unigenes, 22,188 (33.6%) were 201 to 500 bp; 13,444 (20.4%) were 501 to 1,000 bp; 10,421 (15.8%) were 1,001 to 1,500 bp; 7,682 (11.7%) were 1,501 to 2,000 bp; 4,956 (7.5%) were 2,001 to 2,500 bp; 2,871 (4.4%) were 2,501 to 3,000 bp; and 4,388 (6.7%) were more than 3,000 bp in length (Fig 1).

### Sequence annotation

**Fig 2:**
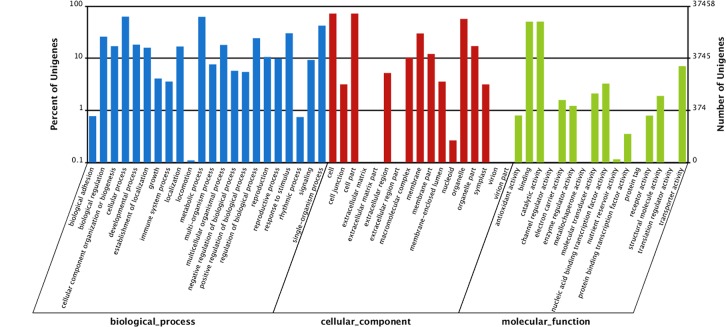
Gene ontology (GO) classification of assembled unigenes.

For annotation of the sequence assembly contigs and unique singletons, all unigenes were searched against the five databases (see Materials and methods). A total of 65,950 unigenes showed significant BLAST hits. Among those unigenes, 47,009 (71.3%) showed significant similarity to known proteins in the NR sequence database of which 31776 (48.2%) were similar to protein in the Swiss-Prot. A total of 51,131 unigenes were annotated in all databases. Based on annotation against NR database, 11,580 (18.4%) unigenes were assigned to gene ontology (GO) terms (Fig 2). The sequences that belonged to the biological process, cellular component and molecular function clusters were categorized into 50 terms. Under the biological process category, the highest sub-category was cellular process (23,555, 16.1%), followed by metabolic process (23,390, 16.0%), single-organism process (15,823, 10.8%) and locomotion (41, <0.1%). Under the cellular component category, cell component (26,966, 25.2%) and organelle component (21,271, 19.9%) represented the majorities, whereas only a few unigenes were assigned to virion (17, <0.1%), virion part (7, <0.1%) and extracellular matrix part (4, <0.1%). Under the molecular function category, catalytic activity (18,927, 42.1%) and catalytic binding (18,878 42.0%) were prominently represented. Furthermore, 2,637 unigenes were involved in transporter activity, whereas only a few unigenes were assigned to protein tag (3), translation regulator activity (3) and channel regulator activity (2).

### Frequency and distribution of different SSR types

Of the 65,950 unigenes generated in the study, 14,547 contained an SSR, 3,350 sequences had more than one SSR, and 1,372 had SSRs of different motifs (compound SSRs). The proportion of EST-SSRs was not evenly distributed. Tri-nucleotide repeat motifs were the most abundant (3,168 or 39.9%) followed by di- (2,987 or 37.6%), mono- (1,189 or 15.0%), hexa- (232 or 2.9%), penta- (199 or 2.5%) and tetra-nucleotide (172 or 2.2%) repeat motifs (Table 1).

**Table 1:** Summary of the number of repeat units in adzuki bean EST-SSR loci.

Repeat motif	No. of repeats								
	4	5	6	7	8	9	10	>10	Total
**Mono-nucleotide (1,189)**									
**A/T**	-	-	-	-	-	-	-	1,177	1,177
**C/G**	-	-	-	-	-	-	-	12	12
**Di-nucleotide (2,987)**									
**AG/CT**	-	-	786	400	251	190	283	134	2,044
**AT/TA**	-	-	176	125	98	65	74	66	604
**AC/GT**	-	-	188	62	44	29	8	7	338
**Others**	-	-	1	-	-	-	-	-	1
**Tri-nucleotide (3,168)**	-								
**AAG/CTT**	-	538	216	162	33	-	-	-	949
**ATC/ATG**	-	275	102	101	13	-	-	-	491
**ACC/GGT**	-	216	79	47	13	-	-	-	355
**AAT/ATT**	-	174	68	62	7	-	-	-	311
**AGC/CTG**	-	177	61	23	16	-	-	-	277
**Others**	-	575	152	46	11	0	1	-	785
**Tetra-nucleotide (172)**									
**AAAG/CTTT**	-	43	2	-	-	-	-	-	45
**AGAT/ATCT**	-	22	1	-	-	-	-	-	23
**AGCC/CTGG**	-	18	-	-	-	-	-	-	18
**AAAT/ATTT**	-	12	5	-	-	-	-	-	17
**Others**	-	44	25	-	-	-	-	-	69
**Penta-nucleotide (199)**									
**AAAAG/CTTTT**	32	-	-	-	-	-	-	-	32
**AAGAG/CTCTT**	28	-	-	-	-	-	-	-	28
**AAAAT/ATTTT**	23	1	-	-	-	-	-	-	24
**AACAC/GTGTT**	11	1	-	-	-	-	-	-	12
**Others**	94	9	-	-	-	-	-	-	103
**Hexa-nucleotide (232)**									
**AACGGG/CCCGTT**	14	-	-	-	-	-	-	-	14
**AAGATG/ATCTTC**	14	-	-	-	-	-	-	-	14
**AACACC/GGTGTT**	11	-	-	-	-	-	-	-	11
**ACCAGC/CTGGTG**	11	-	-	-	-	-	-	-	11
**AAGGAG/CCTTCT**	10	-	-	-	-	-	-	-	10
**Others**	172	-	-	-	-	-	-	-	172
**Total**	420	2,105	1,862	1,028	486	284	366	1,396	7,947
**%**	5.29	26.49	23.43	12.94	6.12	3.57	4.61	17.57	100

The number of SSR repeats ranged from 4 to 24, with five repeats being the most abundant, followed by six and seven repeats as the next most abundant. Motifs with more than 16 repeats were rare (1.8%). Among nucleotide repeats, the A/T (99.0%) was the most abundant. The other six major motifs were AG/CT (68.4%), AAG/CTT (30.0%), AAAG/CTTT (26.2%), AAAAG/CTTTT (16.1%), and AACGGG/CCCGTT (6.0%).

### Development of polymorphic EST-SSR markers in adzuki bean

A total of 7,947 EST-SSR markers were able to be developed from the 14,547 SSR-containing unigene sequences (S2 Table). Five hundred SSR markers were randomly chosen to test amplification and informative nature by analyzing in a germplasm panel of 32 adzuki bean accessions (S3 Table). Of the 500 markers, 296 (59.2%) produced clear amplicons of the expected size, 56 (11.2%) amplified non-specific products, and 148 (29.6%) failed to amplify DNA product. Of the successful markers, 38 (12.8%) showed polymorphisms in the 32 adzuki bean accessions (S3 Table). Based on this rate of polymorphism, 600 polymorphic EST-SSR markers were expected from the 7,947 EST-SSR primer pairs we designed. Among the polymorphic markers were 3, 15, 5, 2 and 14 were di-, tri-, tetra-, penta- and hexa-nucleotide repeats marker. The polymorphism ratio of the EST-SSR markers with di-, tri-, tetra-, penta-, and hexa-nucleotide repeats were 7.9%, 39.5%, 13.2%, 5.3% and 36.8%, respectively. Most of the polymorphic EST-SSR was 4 repeat (39.5%), followed by 7 (21.1%), 8 (15.8%), 6 (10.5%), 5 (7.9%), 9 (2.6%) and 11 (2.6%), respectively. Meanwhile, most of the monomorphic EST-SSR was 4 repeat (33.3%), followed by 6 (25.9%), 7 (16.9%), 5 (10.6%), 9 (4.3%), 10 (3.5%), 8 (3.1%) and 11 (2.4%), respectively. The polymorphic markers detected 86 alleles in total with allele numbers varying between 2 and 4 and with a mean of 2.3 per marker.

### Phylogenetic analysis of the cultivated adzuki bean accessions

The 38 polymorphic EST-SSRs developed in this study were used to assess the genetic diversity and the genetic relationships between the 32 adzuki bean accessions. These accessions were from across the geographic crop distribution in China. Observed heterozygosity (H_o_) varied from 0 to 0.2548, with an average of 0.0312 (Table 2). Gene diversity (H_e_) ranged from 0.1460 (Az3756) to 0.6890 (Az67609) with an average of 0.4790 (Table 2). PIC values ranged from 0.0620 (Az66354) to 0.3980 (Az50799), with an average of 0.2573 (Table 2).

**Table 2:** Characteristics of the polymorphic SSR markers in 32 adzuki bean accessions.

Markers	Na^a^	He^b^	PIC^c^
**Rb9711**	3	0.1250	0.3589
**Rb19803**	3	0.2258	0.3740
**Rb21640**	6	0.1923	0.4484
**Rb197191**	2	0.0000	0.3197
**Rb27353**	5	0.0938	0.4582
**Rb18775**	3	0.0357	0.2457
**Rb22422**	3	0.0313	0.0854
**Rb19149**	3	0.1250	0.3750
**Rb16594**	3	0.0667	0.2688
**Rb28852**	3	0.1875	0.3047
**Rb26585**	3	0.0323	0.2765
**Rb24756**	7	0.1563	0.5718
**Rb93021**	3	0.0313	0.2713
**Rb214492**	3	0.1613	0.2174
**Rb17362**	2	0.0385	0.0733
**Rb25883**	2	0.0625	0.0587
**Rb29169**	3	0.0938	0.3688
**Rb9589**	3	0.0323	0.2765
**Rb28613**	3	0.1290	0.2340
**Rb205762**	4	0.0400	0.2356
**Rb19506**	3	0.0313	0.2125
**Rb9818**	4	0.1563	0.2557
**Rb19643**	3	0.1250	0.3740
**Average**	2.3	0.0945	0.2898

^a^Number of observed alleles.

^b^Expected heterozygosity.

^c^Polymorphic information content.

**Fig 3:**
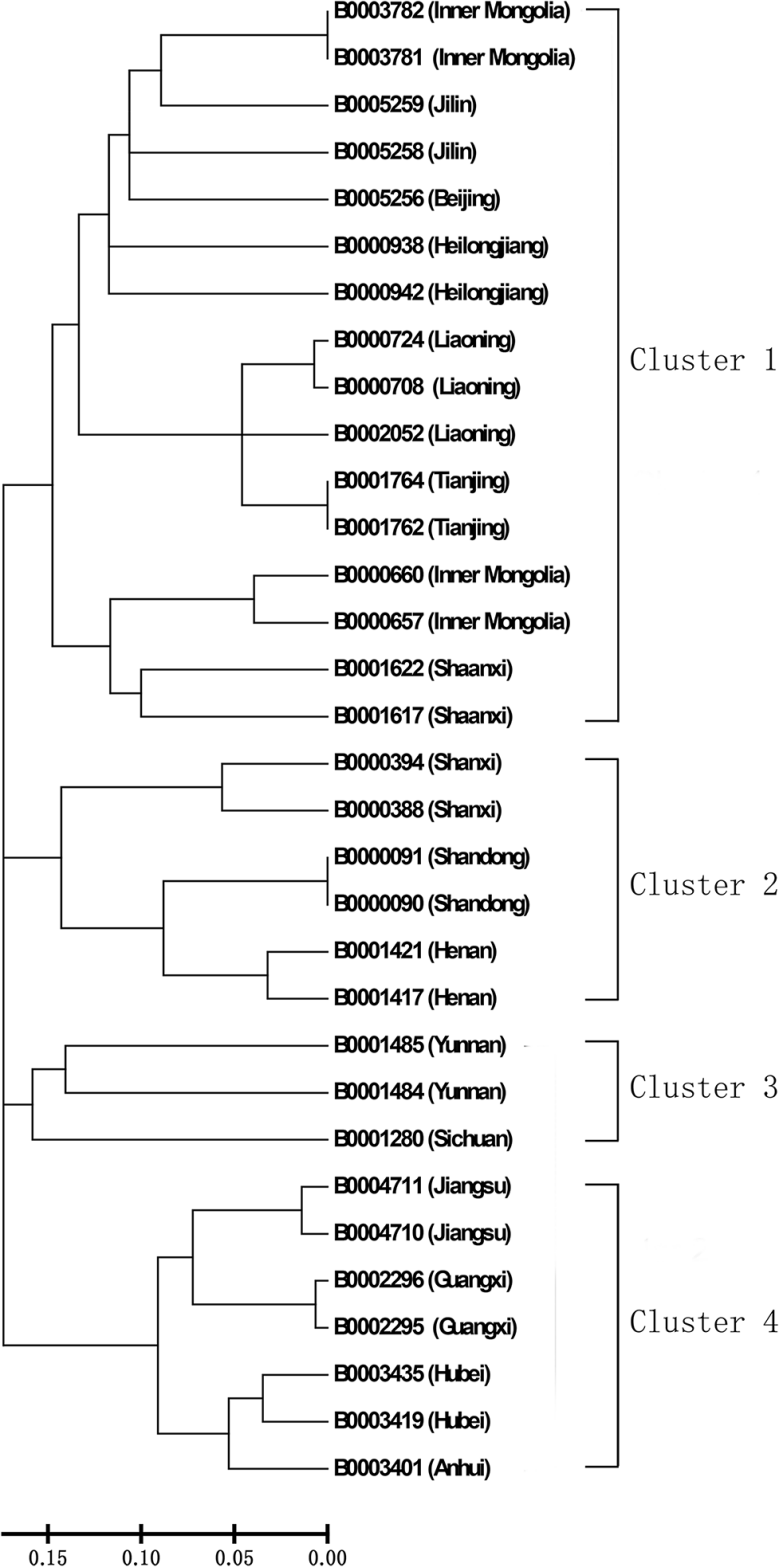
NJ dendrogram based on share allele distance showing genetic relationships among 32 adzuki bean accessions.

NJ tree analysis, based on shared allele distance, grouped the 32 adzuki bean accessions into four main clusters (Fig 3). Both clusters I and II were composed of accessions from northern China, including Jilin, Heilongjiang, Liaoning, Shan’anxi, Shanxi, Shandong, Henan, Beijing, Tianjing, and Inner Mongolia. Clusters III and IV were mostly composed of accessions from southern China, including Yunnan, Sichuan, Jiangsu, Guangxi, Hubei, and Anhui provinces. These results showed an association between genetic relationship of the adzuki bean and geographical origin.

### Gene functions of the unigene sequences containing polymorphic EST-SSRs

To determine the putative functions of the 38 polymorphic EST-SSRs, the unigene sequences used for the development of these markers were subjected to BLASTn analysis against a non-redundant database of legume sequences (S4 Table). The results showed that most of the sequences were similar to hypothetical protein-encoding genes from common bean (*Phaseolus vulgaris* L.), soybean (*Glycine max* (L.) Merr.), and alfalfa (*Medicago truncatula* Gaertn.). A few examples among the positive hits were genes for cyclic nucleotide-gated ion channel 4-like protein, lipase ROG1-like protein, ferritin, RING-H2 finger protein, UTP-glucose-1-phosphate uridylyltransferase, a serine/threonine protein kinase, a zinc finger CCCH domain-containing protein and a transcription factor.

## Discussion

*De novo* transcriptome sequencing has proven to be a crucial tool in many organisms and also an effective way to develop molecular markers [12, 13, 24, 25]. In legumes plants, whole genome sequence of Medicago [26], *Lotus japonicus* [27], soybean [28], pigeonpea [29], chickpea [30], common bean [31], mung bean [32], and adzuki bean [33] has been reported. In adzuki bean, although about a draft genome covering 75% of the estimated genome size has been sequenced and 143,113 SSRs were detected [33], very few transcriptome sequences and EST-SSR markers are developed for this crop [4]. In this study, more than 112 million sequence reads from adzuki bean transcriptome were obtained, representing approximately 20× sequencing depth of the adzuki bean genome. The N50 length of the unigenes was 1,889 bp with the average length of 1,213 bp which are longer than those reported for transcriptome of mung bean (average length of 874 bp, N50 = 1,563 bp) [34], common bean (813 bp and N50 of 1,449 bp) [35], and chickpea (average length of 1,065 bp and N50 length of 1,653 bp) [36]. This suggests the high quality of our adzuki bean transcriptome sequences.

In this study, Illumina sequencing provided a great advantage over traditional sequencing and generated new high-throughput data for transcriptomics and EST-SSR at a low cost and high quality. Transcriptome sequencing is a promising method for marker analysis in species that have no reference genome [37]. Here, we showed that transcriptome sequencing is a very useful method for unigene discovery and marker development in the adzuki bean.

Our work complements a transcriptome analysis using Sanger sequencing of adzuki bean leaf cDNA library from the Japanese adzuki bean cultivar ‘Erimo-shouzu’ that resulted in the development of 1,429 EST-SSR primer pairs [4]. Fifty genomic SSRs were designed and screened against five natural populations of wild adzuki bean, eight primer pairs (16%) showed clear differences between complex and wild populations [3], which was higher than the success rate of EST-SSR development in this study. This difference show that 1) the polymorphism between cultivated and wild adzuki bean is higher than these between cultivated adzuki bean and 2) the polymorphism of genomic SSR markers is higher than EST-SSR markers.

In this study, the tri- (39.9%) and di-nucleotide (37.6%) repeats were the most abundant SSR motifs in adzuki bean transcriptomes. Similar results were reported in other related legume species, including cowpea [38] and chickpea [7]. However, the results were different from mung bean, a closely related species of adzuki bean, which mono- and tetra-nucleotide repeats were the most abundant repeat found in transcriptome sequences (36.2% and 21.4%, respectively) [34]. Only a few previous studies on development of EST-SSR markers in plants gave attention to mono-nucleotide repeats [39]. This type of repeat can increase the number of EST-SSR markers. In mung bean, mono-nucleotide based EST-SSR markers also showed a high polymorphism rate [34]. In our work, 7 mono-nucleotide SSR markers were developed and validated in which all of them showed good amplification but no polymorphism (S3 Table). This result is in line with the result in in common bean that amplification rate of EST-SSR markers based on mono-nucleotide repeats was high and even higher than those based on genomic sequences [39]. Therefore, more attention should be given to mono-nucleotide repeats as sources of SSR markers in the future.

The proportions of di- and tri-nucleotide repeats in adzuki bean transcript sequences were also very close. This is similar to the report in mung bean in which proportions of di- and tri-nucleotide repeats were 13.8% and 14.6%, respectively [34]. Previously, the number of (AG)n and (AC)n motif loci per haploid genome has been estimated to be 3,500 and 2,100, respectively [3]. In this study, we found 656 (AG)n and 78 (AC)n motif loci in the transcriptome sequences, accounting for 22.0% and 2.6% of di-nucleotide motifs, respectively. These data indicate that the (AG)n motifs are rich marker resources in the adzuki bean genome and transcriptome. The most common di-nucleotide repeats found in this study were AG/CT followed by AT/TA. The most common tri-nucleotide repeats were AAG/CTT, followed by ATC/ATG. Similar results were previously reported in adzuki bean [33] and other legumes such as mung bean [34], common bean [35, 40], and faba bean [41]. The most common SSR motifs in adzuki bean ESTs were compared with those of common bean, mung bean, adzuki bean, soybean, medicago and lotus (S5 Table). In these six legume crops, the highest numbers of occurrences of di-nucleotide repeats were from the AG, CT, AT, TA, GA and TC repeats. The highest numbers of tri-nucleotide repeats were from AAG, CTT, GAA, TTC, TCT and AGA repeats. The highest numbers of tetra-nucleotide repeats were from AAAT, ATTT, TTTA and AAAG repeats. The highest numbers of occurrences of penta-nucleotide repeats were from AAAAT, TTTTA, AAAAG and CTTTT repeats. The amplification rate of the adzuki bean EST-SSRs developed in our study (59.3%) is much lower than that of the adzuki bean EST-SSRs developed using Sanger sequencing technology (91.3%) [4]. Low amplifiable rate in our study may stem from (i) large intron between primers [42], (ii) unrecognized intron splice sites that can disrupt priming sites [43], and (iii) sequencing error [44]. Nonetheless, higher amplification rate may be obtained if lower annealing temperature and/or gradient PCR are applied.

Compared to the EST-SSRs developed for other *Vigna* crop including mung bean [34,45] and cowpea [38], the discriminating power, as determined by PIC value, of the adzuki bean EST-SSRs developed in this study (average 0.26; Table 2) is less than that of mung bean EST-SSRs (average 0.34) [34] and cowpea (average 0.53) [38]; however, the adzuki bean germplasms used for allelic diversity analysis in our study were from various geographical regions of China. This suggested that the germplasm diversity used in this study possesses low genetic diversity. The low PIC values of our EST-SSRs suggested that the genetic sequences used for developing those markers are highly conserved in the adzuki bean germplasms used in this study. The low PIC values also suggested that these EST-SSRs may not be suitable for genetic fingerprinting in highly genetically related adzuki bean germplasms. Nonetheless, the polymorphic EST-SSRs were able to classify Chinese adzuki bean germplasms of different geographical origins (Fig 3). With an exception, the germplasms from the same province were clustered to together by the EST-SSRs. A similar result was reported in a large set of germplasm (375 accessions) analyzed by genomic SSR from various legumes [46]. This suggested that adzuki bean cultivars in China were selected and improved for a long time in certain environments, and thus those cultivars specifically adapted to such environments. However, due to the lack of pedigree information of the adzuki bean germplasms used in this study, we were not able to find genetic relationship among adzuki bean accessions in each sub-cluster. Among the polymorphic markers, the markers of tri- and tetra-nucleotide repeat motifs showed polymorphism more frequency (39.5% and 31.6%, respectively) than markers of other repeat motifs.

BLASTX analysis indicated that 34 (89.5%) of the 38 unigenes containing EST-SSRs in common bean and soybean, could be matched to at least one important proteins in the NCBI Nr protein database. For further study, one can search the candidate genes of interest via association analysis referring to the function of markers in the metabolism pathways. The availability of data for EST has given more emphasis to EST-derived SSRs in recently years. These EST-SSRs belong to the transcribed regions of DNA, and they are more conserved and have a higher transferability rate across species than genomic SSR markers. Although EST-derived SSR markers are generally less polymorphic than genomic SSRs [47], the value of EST-SSRs when compared to genomic SSRs is enhanced by several factors including high transferability, potential to attribute function to genes affecting traits of interest, and the readiness in the identification of SSRs by *in silico* data mining with reduced time, labor and cost. It has been reported that more than 83% of the adzuki bean EST-SSR markers could amplify DNA from eight other *Vigna* species including four major crop species, *V*. *angularis*, *V*. *umbellata*, *V*. *radiata* and *V*. *mungo* [4]. The high transferability makes them a powerful tool to study genome of orphan/underutilized *Vigna* crops which less effort has been devoted to develop molecular markers. Therefore, the adzuki bean EST-SSR markers developed in this study will be useful for comparative genomic and genetic diversity studies. For example, recently, genomic regions associated QTLs for agronomically important traits in adzuki bean has been predicted by comparison between adzuki bean and soybean genomes using SSR markers as tool for translation genomics [33]. In summary, we have generated a large number of high-quality unigene sequences from two Chinese adzuki bean varieties by next-generation sequencing and identified a large number of SSRs. We were able to develop up to 7,947 EST-SRR markers from the unigene sequences, of which random marker validation showed that approximately 60% of the EST-SSR markers were amplifiable with clear and expected product sizes. Although the amplifiable markers showed low polymorphism in the adzuki bean germplasm, they were useful for revealing genetic relationships of the germplasm. Our study showed a nearly twenty-fold increase in the number of possible EST-SSRs identified using next-generation sequencing technology compared to previous EST-SSRs developments in adzuki bean using Sanger sequencing technology. This demonstrates that *in silico* SSR marker development by transcriptome sequencing using NGS is a very efficient approach to increase the number of EST-SSRs for crops with low genomic resources. The new SSR sequences and EST-SSR markers developed in this study will be useful resources for genome research and molecular breeding of adzuki bean and other related *Vigna* species.

## Supporting Information

S1 Table Adzuki bean germplasm used in this study. (DOC)

S2 Table Characteristics of adzuki bean EST-SSR markers developed in this study. (XLS)

S3 Table Primer sequences of 500 EST-SSR markers used for marker validation. (XLS)

S4 Table Putative proteins of 38 unigene sequences containing polymorphic EST-SSRs. (DOC)

S5 Table Most common motifs identified in adzuki bean ESTs and in ESTs of five legume crops closely-related to adzuki bean. (DOC)
