# Supplementary material for: Development and Validation of EST-SSR Markers from the Transcriptome of Adzuki Bean (Vigna angularis)
Source: PLoS One. 2015 Jul 6;10(7):e0131939. doi: 10.1371/journal.pone.0131939 (PMC4492930; doi:10.1371/journal.pone.0131939)
Supplement: S1 Table — (DOC) [file pone.0131939.s001.doc]

**S1 Table. Adzuki bean germplasm used in this study.**

| **Serial No.** | **Accession number** | **Province of origin** | **Habit of growth** | **Seed coat color** |
| --- | --- | --- | --- | --- |
| 1 | B0000090 | Shandong | Half-sprawl | Red |
| 2 | B0000091 | Shandong | Half-sprawl | Red |
| 3 | B0000388 | Shanxi | Erect | Red |
| 4 | B0000394 | Shanxi | Erect | Red |
| 5 | B0000708 | Liaoning | Erect | Black |
| 6 | B0000724 | Liaoning | Erect | Red |
| 7 | B0000938 | Heilongjiang | Half-sprawl | Red |
| 8 | B0000942 | Heilongjiang | Half-sprawl | Red |
| 9 | B0001280 | Sichuan | Half-sprawl | Stripe |
| 10 | B0001417 | Henan | Sprawl | Stripe |
| 11 | B0001421 | Henan | Erect | Stripe |
| 12 | B0001484 | Yunnan | Half-sprawl | Red |
| 13 | B0001485 | Yunnan | Erect | Red |
| 14 | B0001617 | Shananxi | Half-sprawl | Yellow |
| 15 | B0001622 | Shananxi | Half-sprawl | Green |
| 16 | B0001762 | Tianjing | Half-sprawl | Red |
| 17 | B0001764 | Tianjing | Half-sprawl | Red |
| 18 | B0002052 | Liaoning | Sprawl | Red |
| 19 | B0002295 | Guangxi | Half-sprawl | Stripe |
| 20 | B0002296 | Guangxi | Half-sprawl | Stripe |
| 21 | B0003401 | Anhui | Erect | Green |
| 22 | B0003419 | Hubei | Erect | Red |
| 23 | B0003435 | Hubei | Sprawl | Red |
| 24 | B0003781 | Inner Mongolia | Erect | White |
| 25 | B0003782 | Inner Mongolia | Erect | White |
| 26 | B0004710 | Jiangsu | Erect | Red |
| 27 | B0004711 | Jiangsu | Erect | Red |
| 28 | B0005256 | Beijing | Erect | Red |
| 29 | B0005258 | Jilin | Half-sprawl | Red |
| 30 | B0005259 | Jilin | Half-sprawl | Red |
| 31 | B0000657 | Inner Mongolia | Erect | Red |
| 32 | B0000660 | Inner Mongolia | Erect | Red |

Note: these accessions were from National Center for Crop Germplasm Resources Preservation of China.
