# Supplementary material for: Development and Validation of EST-SSR Markers from the Transcriptome of Adzuki Bean (Vigna angularis)
Source: PLoS One. 2015 Jul 6;10(7):e0131939. doi: 10.1371/journal.pone.0131939 (PMC4492930; doi:10.1371/journal.pone.0131939)
Supplement: S4 Table — (DOC) [file pone.0131939.s004.doc]

**S4 Table. Putative proteins of 38 unigene sequences containing polymorphic EST-SSRs.**

| **Primer Name** | **Organism**  **(*Phaseolus vulgraris*)** | **GenBank (Accession No.)** | **Organism**  **(*Glycine max* or** ***Medicago truncatula*)** | **GenBank (Accession No.)** |
| --- | --- | --- | --- | --- |
| **Az12487** | Hypothetical protein | XM_007161466.1 | No hit | - |
| **Az13274** | Cyclic nucleotide-gated ion channel 4-like protein | KF033464.1 | Cyclic nucleotide-gated ion channel 4-like protein | XM_003526153.2 |
| **Az13749** | Hypothetical protein | XM_007148289.1 | No hit | - |
| **Az14762** | Hypothetical protein | XM_007135390.1 | No hit | - |
| **Az14987** | |  |  | | --- | --- |   Hypothetical protein | XM_007138354.1 | Lipase ROG1-like protein | XM_003523711.2 |
| **Az14989** | Hypothetical protein | XM_007163513.1 | Ferritin | JX030022.1 |
| **Az15781** | Hypothetical protein | XM_007137591.1 | RING-H2 finger protein | XM_003523725.2 |
| **Az04940** | Hypothetical protein | XM_007137264.1 | UTP-glucose-1-phosphate uridylyltransferase | XM_003604025.1 |
| **Az04794** | No hit | - | No hit | - |
| **Az03756** | Hypothetical protein | XM_007162796.1 | No hit | - |
| **Az50518** | Hypothetical protein | XM_007148528.1 | Zinc finger CCCH domain-containing protein | XM_006598081.1 |
| **Az50799** | Hypothetical protein | XM_007163620.1 | No hit | - |
| **Az14075** | Hypothetical protein | XM_007135326.1 | CCAAT/enhancer-binding protein | XM_003529802.2 |
| **Az14076** | Hypothetical protein | XM_007149354.1 | Transcription factor bZIP112 | XM_003543176.2 |
| **Az13907** | Hypothetical protein | XM_007143650.1 | GATA transcription factor 8-like | XM_003556186.2 |
| **Az23714** | Hypothetical protein | XM_007161466.1 | No hit | - |
| **Az18280** | Hypothetical protein | XM_007157564.1 | No hit | - |
| **Az18293** | No hit | - | No hit | - |
| **Az56917** | Hypothetical protein | XM_007134508.1 | Serine/threonine-protein kinase STN8 | XM_003548281.2 |
| **Az07522** | Hypothetical protein | XM_007152422.1 | No hit | - |
| **Az66354** | Hypothetical protein | XM_007157771.1 | Ras GTPase-activating protein-binding protein | XM_003607762.1 |
| **Az07233** | Hypothetical protein | XM_007137264.1 | UTP-glucose-1-phosphate uridylyltransferase | XM_003604025.1 |
| **Az07222** | Hypothetical protein | XM_007161466.1 | No hit | - |
| **Az67609** | Hypothetical protein | XM_007132407.1 | Uncharacterized | XM_003539110.2 |
| **Az67608** | Hypothetical protein | XM_007161987.1 | R3H domain-containing protein 2-like | XM_003554058.2 |
| **Az19671** | Hypothetical protein | XM_007131435.1 | Uncharacterized | XM_006590803.1 |
| **Az00194** | No hit | - | No hit |  |
| **Az62352** | Hypothetical protein | XM_007159696.1 | No hit | - |
| **Az00497** | Hypothetical protein | XM_007163713.1 | Calmodulin-binding transcription activator 4-like | XM_003552731.2 |
| **Az05415** | No hit | - | No hit | - |
| **Az05350** | Hypothetical protein | XM_007149024.1 | Homeobox-leucine zipper protein HOX11-like | XM_003546813.2 |
| **Az00583** | Hypothetical protein | XM_007138322.1 | No hit | - |
| **Az03190** | Hypothetical protein | XM_007134508.1 | Serine/threonine-protein kinase STN8 | XM_003548281.2 |
| **Az24656** | Hypothetical protein | XM_007135390.1 | No hit | - |
| **Az25655** | Hypothetical protein | XM_007140670.1 | No hit | - |
| **Az02643** | Hypothetical protein | XM_007152422.1 | No hit | - |
| **Az24403** | Hypothetical protein | XM_007148528.1 | Zinc finger CCCH domain-containing protein 5-like | XM_006598081.1 |
| **Az04852** | Hypothetical protein | XM_007161466.1 | No hit | - |
