# Supplementary material for: Development and Validation of EST-SSR Markers from the Transcriptome of Adzuki Bean (Vigna angularis)
Source: PLoS One. 2015 Jul 6;10(7):e0131939. doi: 10.1371/journal.pone.0131939 (PMC4492930; doi:10.1371/journal.pone.0131939)
Supplement: S5 Table — (DOC) [file pone.0131939.s005.doc]

**S5 Table. Most common motifs identified in adzuki bean ESTs and in ESTs of five legume crops closely-related to adzuki bean.**

| **Repeat motifs** | **Common bean** | **Mung bean** | **Adzuki bean** | **Soybean** | **Medicago** | **Lotus** |
| --- | --- | --- | --- | --- | --- | --- |
| **Di-** | AG CT  AT TA  (1827) | AG CT AT TA (1073) | AG CT  AT AT  (2648) | AG CT  GA TC  (2192) | AG CT  GA TC  (982) | AG CT  GA TC  (596) |
| **Tri-** | AAG CTT ACC GGT  (936) | GAA TTC TCT AGA (435) | AAG CTT ATC ATG (1440) | TTC AAG TCT CTT AGA GAA (5694) | TTC AAG TCT CTT AGA GAA (6318) | TTC AAG TCT CTT AGA GAA (2104) |
| **Tetra-** | AAAT TTTA  (78) | AAAT ATTT TTTA TAAA (367) | AAAG CTTT AGAT ATCT (68) | AAAC AACA ACAA CAAA TTTG TTGT TGTT GTTT (1552) | AAAT AATA ATAA TAAA TTTA TTAT TATT ATTT (1287) | AAAT AATA ATAA TAAA TTTA TTAT TATT ATTT (378) |
| **Penta-** | AAAAT TTTTA  (20) | AAAAT ATTTT AAAAG CTTTT (94) | AAAAG CTTTT AAAAT ATTTT (55) | AAAAT AAATA AATAA ATAAA TAAAA TTTTA TTTAT TTATT TATTT ATTTT (356) | AAAAT AAATA AATAA ATAAA TAAAA TTTTA TTTAT TTATT TATTT ATTTT (277) | AAATT AATTA ATTAA TTAAA TAAAT TTTAA TTAAT TAATT AATTT ATTTA (202) |
